# Effect of Consuming Salmon Products on Vitamin D Status of Young Caucasian Women in Autumn—A Randomized 8-Week Dietary VISA 2 (Vitamin D in Salmon Part 2) Intervention Study

**DOI:** 10.3390/nu16203565

**Published:** 2024-10-21

**Authors:** Zofia Utri-Khodadady, Dominika Głąbska, Dominika Guzek

**Affiliations:** 1 Department of Dietetics, Institute of Human Nutrition Sciences, Warsaw University of Life Sciences (WULS-SGGW), 159C Nowoursynowska Street, 02-776 Warsaw, Poland; zofia_utri@sggw.edu.pl; 2 Department of Food Market and Consumer Research, Warsaw University of Life Sciences (WULS-SGGW), 159C Nowoursynowska Street, 02-776 Warsaw, Poland; dominika_guzek@sggw.edu.pl

**Keywords:** vitamin D deficiency, 25(OH)D, salmon sausage, smoked salmon, Salmo salar, fish intake, fish intervention, vitamin D bioavailability

## Abstract

Background/Objectives: Young women are often at risk of vitamin D deficiency, while fatty fish can provide significant amounts of it, which is especially important when no vitamin D skin synthesis is possible due to limited sunshine exposure. This study aimed to analyze the impact of increasing the intake of salmon in various forms (smoked salmon, salmon sausages) on vitamin D status of young women in autumn. Methods: The 8-week intervention involved 120 non-obese women, aged 20–25 years. Participants were randomly assigned to one of three groups: smoked salmon (25 g/day), salmon sausage (100 g/day), or a control group. Both intervention products provided approximately 5 µg of vitamin D daily. Serum concentrations of 25(OH)D as well as vitamin D intakes were assessed pre-, mid-, and post-intervention. Results: The median vitamin D intake at baseline was 2.7–3.4 µg/day and did not differ between the groups (*p* > 0.05), while during the intervention, it was highest in the smoked salmon group (*p* < 0.001) and amounted to 7.3 µg/day. While all groups experienced a decrease in 25(OH)D serum concentrations, the decrease was significantly smaller in the salmon sausage group compared to the control group (−4.3 vs. −15.0 nmol/L, *p* < 0.05), and no significant difference was observed between the smoked salmon and control group after 8 weeks (*p* > 0.05). Moreover, in the salmon sausage group, the intervention was more effective among participants with an inadequate vitamin D status at baseline (25(OH)D change after the intervention: −3.0 vs. −5.4 nmol/L, *p* < 0.05; inadequate vs. adequate baseline vitamin D status). Conclusions: Increasing the intake of salmon, and hence of vitamin D, was not enough to maintain the vitamin D status of young women in autumn. It seems that other, not-yet-fully-understood factors, may influence vitamin D absorption and/or metabolism, thereby affecting the outcomes of such interventions indicating that further research is needed. Nevertheless, it may be concluded that increasing salmon sausage intake might aid slow down the natural decline of 25(OH)D in young women in autumn.

## 1. Introduction

Vitamin D is a multipotential nutrient that is proven to be effective in the treatment and prevention of numerous diseases and conditions [1]. Above all, it promotes calcium and phosphorus absorption in the gut ensuring their adequate concentrations are maintained. As a result, it is essential for the growth and remodeling of osteoblasts and osteoclasts [2], leading to a positive correlation between vitamin D status and bone health, as stated in a meta-analysis by Segheto et al. [3]. Other recent meta-analyses indicate that several diseases can be alleviated or prevented with vitamin D supplementation, including type 2 diabetes [4], cancer [5], depression [6], COVID-19 [7], and acute respiratory infections [8]. Therefore, vitamin D can be used as a dietary-based prevention and treatment means.

The main source of vitamin D for humans is the skin synthesis of cholecalciferol from 7-dehydrocholesterol which is a result of UVB radiation (290–320 nm) during sun exposure [9]. However, it is highly dependent on the latitude, the season, and the time of the day, which results in the fact that in countries located in a moderate climate, vitamin D skin synthesis is possible only from April to October [10]. The other vitamin D sources are dietary. It naturally occurs in fish, eggs, some mushrooms, meat, milk and milk products, but it is also added to food products during production and can hence be found in fortified foods such as margarine or breakfast cereals [11]. What should be underlined, however, is that the best (concerning the amount of vitamin D and the serving size ratio) dietary vitamin D source seems to be fish [12]. Nevertheless, the vitamin D content varies significantly depending on the species—while it can amount to high levels such as in the case of eel (*Anguilla anguilla*)—30 µg/100 g, herring (*Clupea harengus*)—19 µg/100 g or salmon (*Salmo salar*)—13 µg/100 g, other fish species such as cod (*Gadus morhua*) or flounder (*Platichthys flesus*) contain almost no vitamin D (1.0 µg/100 g and 0.8 µg/100 g, respectively) [12].

Importantly, while it is often stated that improper vitamin D status is a global problem [13], there is no consensus on the definition of vitamin deficiency. While some authorities, such as the United States (US) National Institute of Health [14] and the United Kingdom Royal Osteoporosis Society, define it as a 25-hydroxyvitamin D (25(OH)D) serum concentration lower than 30 nmol/L (12 ng/mL) [15], others, such as the European Food Safety Authority (EFSA) [16] and the joint expert panel responsible for the Polish recommendations, indicate a higher threshold—namely 25(OH)D serum concentration lower than 50 nmol/L (20 ng/mL) [17]. Therefore, depending on the threshold used in analyses, the prevalence of vitamin D deficiency in a population varies greatly. For instance, the prevalence of vitamin D deficiency based on data from 14 European countries was 13% considering the threshold of 30 nmol/L, and 40.4% for the 50 nmol/L threshold [18]. What should be underscored, however, is that the prevalence of vitamin D deficiency appears to be even higher among young European women [19,20]. Poland is not an exception, as 22% of females were found to have their vitamin D levels lower than 25 nmol/L [21].

To combat vitamin D insufficiency, some recommendations include supplementing the diet with vitamin D supplements throughout the entire year [17] or specifically during autumn and winter months, when the skin synthesis of vitamin D is significantly reduced [15]. However, some studies demonstrate a positive effect of dietary interventions in improving vitamin D status without the use of supplements. For example, incorporating 450 g of salmon weekly into the diet increased the 25(OH)D serum concentration in adults following an energy-restricted diet [22], consuming seafood (mainly fatty fish) three times a week increased 25(OH)D serum concentration in Norwegian individuals [23], and consuming 60 g of vitamin D3-enriched Gouda cheese daily effectively increased 25(OH)D serum concentrations in women in Greece during the winter [24]. On the other hand, some research showed no effect from the studied intervention. For instance, no increase in vitamin D status was observed in overweight/obese adults in Norway even when consuming 750 g of salmon weekly [25], and there was even a decrease in 25(OH)D serum concentration after 4 weeks of consuming 50 g of smoked salmon daily in young Polish women in the Vitamin D in Salmon (VISA) study [26]. This is summarized in the conclusions from the meta-analysis by Lehmann et al. [27], which indicate that consuming fish increases 25(OH)D serum concentration, but the type of fish (fatty or lean) and the length of the intervention play a vital role in its efficacy.

Considering that young females are especially at risk of vitamin D deficiency, as well as the inconclusive intervention studies aiming at improving vitamin D status, the present study aimed to analyze the impact of increasing the intake of salmon on the vitamin D status of young women in autumn, when sunshine exposure is limited. An additional goal of the study was to evaluate various forms of salmon, such as smoked salmon and salmon sausages, which contain significant amounts of vitamin D but differ in form and nutritional value.

## 2. Materials and Methods

### 2.1. General Information

The Vitamin D in Salmon Part 2 Study (VISA 2 study) was conducted in the Dietetic Outpatient Clinic of the Department of Dietetics, Warsaw University of Life Sciences (WULS-SGGW) in Poland. Since the VISA 2 study was a follow-up of the previously conducted VISA study [26] the study participants, as well as the study design were as similar to the VISA study as possible. Nevertheless, the VISA 2 study included two intervention groups and one control group, in comparison to the VISA study, due to the additional comparison of the different forms of salmon (smoked salmon and salmon sausages).

The VISA 2 study was conducted according to the guidelines of the Declaration of Helsinki. It was approved by the Ethics Committee of the Faculty of Human Nutrition and Consumer Sciences of the Warsaw University of Life Sciences (No. 27/2018) and the Ethics Committee of the Central Clinical Hospital of the Ministry of Interior and Administration in Warsaw (No. 2/2021). The study was not recorded on any dedicated trial register platforms. All the study participants provided informed consent for participation in the study and all its procedures, and they were allowed to withdraw at any time.

### 2.2. Studied Group

Study participants for the research were recruited using the convenience sampling procedure. The invitation to take part in the study was announced on university social media, while the snowball effect was also allowed. The inclusion criteria were females, 18–30 years old, living in Warsaw or its surroundings (making it possible to visit the Dietetic Outpatient Clinic of the Department of Dietetics, Warsaw University of Life Sciences (WULS-SGGW) several times during the study) and written informed consent to participate in the study. The exclusion criteria were as follows: pregnancy, lactation, obesity (defined as BMI ≥ 30.0 kg/m^2^), following a vegan or any other fish-excluding diet, using vitamin D supplements at least 3 weeks before the study beginning, fish and/or seafood allergy, diseases which influence vitamin D metabolism, using medications which influence vitamin D metabolism, planned travels to places based below the 40th parallel north and planned solarium ultraviolet radiation exposure during the study time.

In total, 120 women met the inclusion criteria and were randomly assigned to one of three study groups (group 1—Smoked salmon group, group 2—Salmon sausage group, and group 3—Control group) with 40 participants in each group. The random assignment was performed using stratified block randomization, with stratification based on baseline 25(OH)D concentration. This allocation ensured that the baseline mean 25(OH)D concentrations were similar across the groups. Additionally, the baseline mean BMI was comparable across the groups; therefore, no further adjustments to the three study groups were made.

The study participants did not obtain any monetary compensation for taking part in the study. However, as part of the study, they received free medical evaluation of their 25(OH)D serum concentration and body composition (both three times at three time points). Also, participants in the intervention groups were given the intervention products at no cost.

### 2.3. Dietary Intervention

The fish intake was planned to be 175 g of smoked salmon weekly to align with fish intake recommendations, which in most European countries and the USA are approximately 150–300 g weekly [28]. This amount was also stated to be potentially easily incorporated into daily life. The quantity of salmon sausages was adjusted to ensure similar vitamin D intakes in both intervention groups, averaging around 5 µg per day, resulting in a final amount of 700 g of salmon sausages per week. Specifically, the daily vitamin D content in the intervention products was calculated as 4.4 µg in salmon sausages and 5.3 µg in smoked salmon. This amount of vitamin D covers around one-third of the recommended adequate intake in Poland, which is 15 μg [29].

Taking into account the described assumptions, depending on the assigned group, each woman was asked to incorporate into her diet 25 g of smoked salmon (group 1) or 100 g of salmon sausages (2 sausages; group 2) daily or not to change anything in her diet (group 3). Participants from groups 1 and 2 were allowed alternatively to consume twice the daily portion every second day, namely: group 1–50 g of smoked salmon, and group 2–200 g of salmon sausages (4 sausages), if needed. Due to that, it was assessed whether there were differences concerning the frequency of consuming the intervention products daily or every second day between the two intervention groups. Indeed, there were statistically significant differences (*p* = 0.002) between the groups. Participants in the salmon sausage group consumed the intervention product more frequently on a daily basis compared to those in the smoked salmon group (44.5% vs. 18.5%, respectively). Conversely, participants in the smoked salmon group consumed the product more often every second day compared to those in the salmon sausage group (52.5% vs. 11.0%, respectively). The remaining participants did not report a fixed frequency of consumption.

In the case of omitting the salmon/salmon sausage consumption, participants were asked to consume them as soon as they could for the total salmon/sausage intake to be similar among the whole group. Participants were allowed to consume the intervention products as they were, as well as to cook them in water, fry, or bake them. Apart from the intervention, study participants were instructed not to alter their habitual diet regarding fish and any other products. They were asked not to exclude or add fish to their diet in a way that would disrupt their usual eating patterns. Therefore, participants in the control group could incorporate any fish, including salmon, into their diet, while participants in the intervention groups could consume additional fish, including salmon, only if it aligned with their general dietary patterns.

Based on the indication that fasting conditions (i.e., gastric pH = 1) enhance vitamin D bioaccessibility [30], an additional chi-square analysis concerning the frequency of consuming the intervention product on an empty stomach (as part of the first meal of the day) was conducted. However, no differences were seen between the smoked salmon and the salmon sausage group (55% vs. 63%, *p* = 0.714, respectively).

Both the smoked salmon and the salmon sausages that were used for the study were obtained from one producer, one of the leading salmon sellers in Poland (Suempol Polska Ltd.). The provided products were obtained from the same batch in identical sliced trays (smoked salmon) or identical sausage trays (salmon sausages) and modified atmosphere packaging. Both the smoked salmon and the salmon sausages were produced from farmed Atlantic salmon (*Salmo salar*) from Norway. Importantly, Atlantic farmed salmon is a fish species that contains very low levels of mercury and dioxins. It has been classified by the FAO/WHO expert consultation into the first and second groups, respectively, for having the lowest concentrations of these contaminants. This, along with its high concentrations of eicosapentaenoic acid (EPA) and docosahexaenoic acid (DHA), led the FAO/WHO to state that the benefits of consuming Atlantic farmed salmon outweigh the potential risks associated with the contaminants [31]. Due to the large variations in the content of vitamin D in different salmons [32], the vitamin D content was measured in the products by a leading vitamin laboratory in Europe—Eurofins Vitamin Testing Denmark (EN 12821: 2009-08 [33], LC-DAD, accredited methodology no. 581). The nutritional value of the used smoked salmon and salmon sausages is presented in Table 1.

The ingredients of the smoked salmon used in the intervention were Atlantic salmon (*Salmo salar*) and salt, while of the salmon sausages were Atlantic salmon (*Salmo salar*) 85%, water, salt, spice extracts, glucose, flavors, potato starch, plant fibers, pea starch.

Because pH appears to influence vitamin D bioavailability and/or absorption [30], the pH of the smoked salmon and the salmon sausages used in the intervention was also assessed. The pH measurements were conducted using a potentiometric method with a hand-held pH meter (Testo AG 205, Lenzkirch, Germany) calibrated against two buffers (pH = 4.01, pH = 7.00). Each product was analyzed in at least five repetitions. The median pH of the salmon sausages was slightly higher than that of the smoked salmon (6.49 vs. 6.15; *p* = 0.004; U Mann–Whitney test), indicating that the smoked salmon was slightly more acidic. However, analyses show that there are no differences in vitamin D stability at pH values between 5 and 8, suggesting that the pH of the used products should not have been a confounding factor [34].

To increase the intervention compliance and to ensure that study participants consume fish products with the same nutritional value, the products were provided to the participants and financed as part of the intervention. Participants were asked to pick the products up every two weeks at the Dietetic Outpatient Clinic of the Department of Dietetics, Warsaw University of Life Sciences (WULS-SGGW), where the study was conducted. Moreover, to minimize the bias associated with forgetting to consume the given products, they completed a control sheet daily, reporting the products consumed as part of the intervention. During the study, every time the participants collected the packages of the intervention products, they handed in the control sheet and were asked whether they had managed to consume the products, at what frequency (daily or every second day) they consumed them, how they liked them, and how they were feeling concerning the intervention.

### 2.4. Measurements

#### 2.4.1. Vitamin D Status

The study was divided into 2 parts, 4 weeks each, to verify the status of the participants in the middle of the intervention and after the whole intervention. Therefore, the participants had their vitamin D status measured three times—before the intervention (at baseline), 4 weeks after the beginning of the intervention (in week 5), and after 8 weeks of the intervention (in week 9). Likewise, the anthropometric measurements were performed, and the participants filled in the questionnaire assessing vitamin D intake also before, during (in week 5), and after the intervention (in week 9).

Vitamin D status was assessed based on the total 25(OH)D serum concentration. Venous blood samples were drawn by specialized nurses at four certified medical analysis laboratories in Warsaw (ALAB Laboratoria Ltd.), and the participants were able to choose their preferred location. The participants did not have to be in a fasting state for the blood collection. The total 25(OH)D serum concentrations were assessed on an Abbott’s ALINITY I analyzer using the direct chemiluminescence method. Because the definitions of vitamin D sufficiency differ between authorities [35], the obtained results of the total 25(OH)D serum concentrations were compared to two different reference value ranges, as follows:

- <50 nmol/L—inadequate, 50–250 nmol/L—adequate, >250 nmol/L—potentially toxic [15,16];
- <75 nmol/L—inadequate, 75–250 nmol/L—adequate, >250 nmol/L—potentially toxic [17].

Not to influence the results of the study (especially concerning the intake of vitamin D-rich foods and the intake of vitamin D supplements) as well as to increase adherence, the participants were provided with their 25(OH)D serum concentration results only after the study finished.

#### 2.4.2. Anthropometric Measurements

Anthropometric measurements were conducted using ACCUNIQ BC720 (Selvas Healthcare, Daejeon, Republic of Korea), which enabled the measurement of body weight with a calibrated scale (accuracy: ± 0.5 cm, range: 10–270 kg) and height with a calibrated ultrasound stadiometer (accuracy ± 0.1 cm, range: 50–220 cm), as well as the estimation of soft lean mass (SLM), total body water (TBW), fat mass (FM), free-fat mass (FFM) and skeletal muscle mass (SMM). Body weight and height measurements were performed according to commonly accepted protocols [36], and the body mass index (BMI) was calculated using the Quetelet equation [37]. The ACCUNIQ BC720 makes the body composition estimations based on bioelectrical impedance measurements (range: 100–950 Ω), with eight electrodes, using six different frequencies (1, 5, 50, 250, 550, 1000 kHz), which are then recalculated by the built-in program into the different body parts weights.

The study participants were instructed on how to prepare for the measurement, including abstaining from alcohol for 24 h before the test, avoiding intensive physical activity on the day of the examination, refraining from drinking fluids for 3 h prior, not eating for 4 h before, urinating just before the measurement, wearing light clothing without metal parts (such as a wireless sports bra), and removing any metal items (e.g., jewelry, watches). For the measurements, each participant came in individually to ensure privacy, and, before the measurements, it was made sure that they were correctly prepared concerning the above-written instructions. Participants were then asked to remove heavy clothing such as jumpers, etc., as well as socks or thighs, to step on the device barefoot, place their feet on the electrodes (visible metal parts), and stand still. After the weight and height measurements were conducted, the participants were asked to take the electrode handles so that all fingers touched the electrodes (visible metal parts). The device transmitted a current of 180 μA, which enabled the body composition measurements. After the measurements, the participants were asked to step off the device and dress. The assumed clothing weight was 0.5 kg, which was automatically subtracted by the built-in program.

Participants were provided only with their weight and height results during the measurement, while all the body composition results were given to them after the study finished to increase adherence. The anthropometric measurements were carried out in the Dietetic Outpatient Clinic of the Department of Dietetics, Warsaw University of Life Sciences (WULS-SGGW).

### 2.5. Questionnaire

To assess vitamin D intake, all participants filled in an adjusted version of the Vitamin D Estimation Only—Food Frequency Questionnaire (VIDEO-FFQ) which had previously been validated among young women aged 20–30 years [38]. Since salmon sausages are not included in the questionnaire, an additional question regarding the intake of salmon sausages was included in the questionnaire to assess vitamin D intake. To calculate the daily vitamin D intake, the given formula [38] was adjusted by adding the vitamin D intake from the salmon sausages to the sum—assuming that 100 g of salmon sausages (daily portion) provided 4.41 μg of vitamin D (as shown in Table 1). Moreover, the vitamin D content in the specific smoked salmon used in the intervention was 21.32 μg/100 g (as shown in Table 1), whereas the original version of the questionnaire [38] assumed a mean content of 15 μg/100 g. Therefore, the actual measured vitamin D content (21.32 μg/100 g) was used in the formula to calculate the daily vitamin D intake for all smoked salmon portions consumed by participants in the smoked salmon intervention group. The questionnaire was prepared in Google Forms, while the link to it was sent to the participants via email.

For the analysis, fish intake, fish product intake, and the total fish and fish product intake were calculated based on the questions and answers from the adjusted version of the VIDEO-FFQ. Just like in the case of the VIDEO-FFQ [38], fish (fresh and smoked) were grouped into these with a high vitamin D content (≥15 µg/100 g; salmon (*Salmo salar*), rainbow trout (*Oncorhynchus mykiss*), herring (*Clupea harengus*), and eel (*Anguilla anguilla*)), with a medium vitamin D content (2.1–8.0 µg/100 g; halibut (*Hippoglossus hippoglossus)*, mackerel (*Scomber scombrus*), brook trout (*Salvelinus fontinalis*), sole (*Solea solea*), and tuna (*Thunnus*)) and with a low vitamin D content (≤1.0 µg/100 g; cod (*Gadus morhua*), flounder (*Platichthys flesus)*, plaice (*Pleuronectes platessa*), pollock (*Gadus chalcogrammus*), hake (*Merluccius merluccius*), perch (*Perca fluviatilis*), zander (*Sander lucioperca*), and pike (*Esox Lucius*)) (see Supplementary Materials).

Because some participants in the intervention groups reported improved dermatological health (complexion and hair) during the course of the study, an additional open-ended question about any observed health outcomes was asked to all participants at the end of the study. Due to the high number of answers concerning dermatological health, all answers indicating dermatological health improvements (improved complexion, more elastic and moisturized skin, fewer pimples, faster healing of skin lesions, enhanced hair condition, shiny hair, stronger nails, etc.) and dermatological health deterioration (deterioration of complexion, appearance of acne, non-healing pustules, weakening of hair condition, hair loss, etc.) were grouped and the frequency of reporting dermatological health improvement vs. deterioration was compared between the groups.

Furthermore, each participant was asked whether they had any difficulties consuming the provided products, such as finding them repetitive or tiresome, and if they would have liked the intervention to continue longer.

### 2.6. Study Course

**Figure 1:**
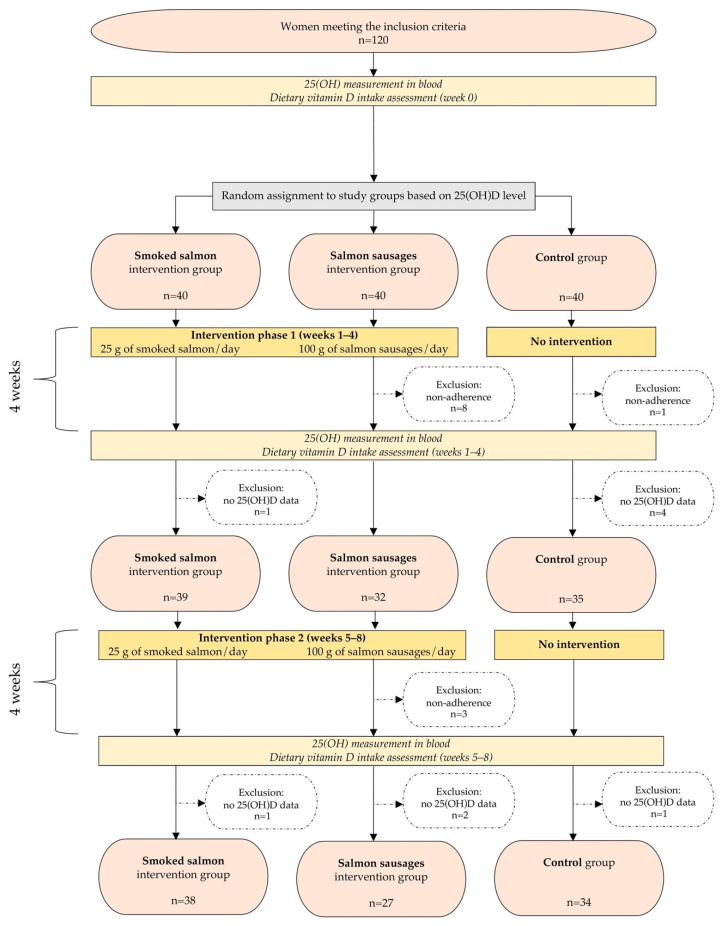
Course of the study.

The course of the study is presented in Figure 1. During the study, some participants withdrew, while others were excluded due to non-adherence to the dietary intervention or failure to attend the blood draw. The total drop-out rate was 17.5%, and finally, 99 participants were included in the analyses.

#### 2.6.1. Wash-Out Before Study Beginning

Because vitamin D supplementation is widely recommended in the Polish population [17], a 3-week wash-out was planned for those who had supplemented their diet with vitamin D before. In total, 20 out of the 99 participants (20.2%) declared to have supplemented their diet with vitamin D in the form of a supplement, fish oil, or a multivitamin 3 weeks before the study began. Despite the wash-out, their initial 25(OH)D serum concentration was higher than among those who did not use such supplements (80.2 nmol/L vs. 61.2 nmol/L; *p* = 0.003). However, the change in 25(OH)D during the intervention was not influenced by whether participants had been taking vitamin D supplements before the study (*p* = 0.243; *p* = 0.522; *p* = 0.536; change w0-w5, w9-w5, and w0-w9, respectively, comparing individuals who took and did not take vitamin D supplements 3 weeks before the study). Therefore, it was assumed that the wash-out time was correctly planned.

#### 2.6.2. Study Time

As vitamin D status changes throughout the year, all participants were enrolled in and finished the study simultaneously. The intervention was conducted in autumn and lasted from 24 October to 18 December 2022, identical to the day in the VISA study in 2018 [26]. Considering the temperatures during the VISA study in 2018 and the current VISA 2 study, they were alike too—the mean temperature in the Masovian region was 10 °C in October 2018 and 11 °C in October 2022, 4 °C in November 2018 and November 2022, and 2 °C in December 2018 and 1 °C in December 2022 [39,40]. Likewise, the reason for choosing this time of the intervention was the fact that in countries such as Poland, vitamin D synthesis in the skin, being the major source of the vitamin, is only possible from April to October [10]; hence, it was decided to conduct the study after this period.

### 2.7. Statistical Analyses

The accepted level of statistical significance was *p* ≤ 0.05. To assess the normality of distribution, the Shapiro–Wilk test was used. The results are presented as mean ± SD and median with a range of percentiles (P25; P75), while the values used in the discussion depended on the normality of the distribution: mean ± SD for normal distributions and median for non-normal distributions.

The sample size required for the analyses was calculated based on the population of females aged 19–30 in the Masovian Voivodeship (318,200, according to the Central Statistical Office of Poland [41]). Assuming 25.9% as the obesity rate for this group [42], the non-obese population was determined to be 235,786. A 95% confidence level, 10% margin of error, and 20% prevalence of low vitamin D concentrations (<25 nmol/L) [21] were applied, resulting in an estimated required sample size of 61. The final sample of 99 females was therefore considered sufficient.

For group comparisons, the U Mann–Whitney test was used (in case of two groups and non-normal distribution) or the analysis of variance (ANOVA) followed by Tukey’s Honest Significant Difference (HSD) test for the post hoc analysis (in case of three or more groups and data of normal distribution) or the Kruskal–Wallis analysis of variance (ANOVA) followed by the Dunn–Sidak-Corrected Fisher (DSCF) test for the post hoc analysis (in case of three or more groups and data of non-normal distribution). The chi-square test was used to analyze the differences between the groups concerning categorical variables, whereas the chi-square test with Yates’ correction was taken into account when needed.

The analyses were conducted in Statistica 13.3 (Statsoft Inc., Tulsa, OK, USA) and Jamovi (The Jamovi project, version 2.3, Sydney, Australia) [43] software.

## 3. Results

### 3.1. Anthropometric Characteristics of the Participants

Table 2 presents the detailed anthropometric characteristics of the two intervention groups and the control group at baseline. Most of the anthropometric characteristics of the participants did not differ between the three studied groups (*p* > 0.05) apart from the age in the control group and the smoked salmon group (23 vs. 21 years; *p* = 0.041 for pairwise comparison) and the body fat mass, which was higher in the salmon sausages group than in the smoked salmon group (27.4% vs. 23.7%; *p* = 0.012 for pairwise comparison). The weight change from baseline to after 4 weeks of intervention, from after 4 weeks of intervention to after 8 weeks of intervention, and from baseline to after 8 weeks of intervention, did not differ among the three groups either (*p* > 0.05).

### 3.2. Fish Intake Throughout the Study

The comparison of the weekly intake of fish and fish products among the participants from the different study groups at baseline, during the 4 first weeks and the last 4 weeks of the intervention is presented in Table 3. At baseline, the weekly intake of fish and fish products amounted to 78.8–128.0 g and did not differ between the groups (*p* > 0.05). However, more participants from the salmon sausage intervention group complied with the recommendation to consume at least 150 g of fish and fish products weekly than participants from the smoked salmon and the control group (48.1 vs. 21.1 and 20.6%; *p* = 0.027; data not presented in tables). During the intervention, the weekly intake of fish and fish products was highest among participants from the salmon sausage group followed by those from the smoked salmon group, and lowest among those from the control group (w1-w4: 712.0 vs. 222.0 vs. 64.2 g/week, *p* < 0.001 and w5-w8: 711.0 vs. 210.0 vs. 58.3 g/day, *p* < 0.001). These differences result from the fact that participants from the salmon sausage group consumed 100 g of salmon sausages daily (corresponding to 700 g/week) within the applied intervention, while participants from the smoked salmon group consumed 25 g of smoked salmon daily (corresponding to 175 g/week) within the applied intervention. During the study, all participants from both intervention groups complied with the recommendation to consume at least 150 g of fish and fish products weekly, while in the control group, it was 11.8% (*n* = 4) during the first 4 weeks of the intervention and 17.6% (*n* = 6) during the last 4 weeks of the intervention (data not presented in tables).

A detailed analysis of the daily intake of fish species grouped based on their vitamin D content, the daily intake of fish products, and the total daily fish and fish products intake across the different study groups at baseline, during the 4 first weeks and the last 4 weeks of the intervention is presented in Supplementary Table S1.

### 3.3. Vitamin D Intake Throughout the Study

The comparison of the daily intake of vitamin D among the participants from the different study groups at baseline, during the 4 first weeks and the last 4 weeks of the intervention is presented in Table 4. Whereas the baseline daily vitamin D intake did not differ between the groups at baseline (*p* > 0.05), it did during the intervention with participants from the smoked salmon group consuming the highest amounts and those from the control group consuming the lowest amounts of vitamin D (w1-w4: 7.3 vs. 6.8 vs. 2.3 µg/day; w5-w8: 7.3 vs. 6.5 vs. 2.3 µg/day; *p* < 0.001). However, the comparison between the groups considering vitamin D intake excluding vitamin D from the intervention products did not reveal any differences (*p* > 0.05), which indicates that the habitual vitamin D intake (without vitamin D from the intervention products) was not a factor which might have influenced the obtained results.

### 3.4. Vitamin D Status (Total 25(OH)D Serum Concentration) Throughout the Study

Participants’ total 25(OH)D serum concentrations and their changes at baseline, after 4 weeks, and after 8 weeks of the intervention are presented in Table 5. The mean baseline serum concentration in the whole studied population amounted to 65.1 ± 24.9 nmol/L and did not differ between the groups (*p* > 0.05). Surprisingly, the serum concentration did not differ between the groups after 4 and 8 weeks of the intervention either (*p* > 0.05). A decrease in the 25(OH)D concentration was observed after 4 and 8 weeks of intervention in all groups. However, the declining *p*-value from *p* = 0.947 at baseline to *p* = 0.130 after 8 weeks of the intervention suggests that the duration of the observation time may have been too short to reveal a statistically significant difference. Nevertheless, differences between the groups were observed concerning the changes in 25(OH)D serum concentration (*p* < 0.05) with the biggest decrease in the control group and the smallest decrease in the salmon sausage intervention group. The post hoc analysis indicated that for the changes from w0 to w5 and from w5 to w9, the differences between the groups concerned only the control group and the salmon sausage intervention group (*p* = 0.019 and *p* = 0.017, respectively), while for the change from w0 to w9, differences were observed comparing the control group with the salmon sausage group (*p* < 0.001), as well as when comparing the smoked salmon group and the salmon sausage group (*p* = 0.034). No difference was observed when comparing the 25(OH)D serum concentration change from w0 to w9 in the smoked salmon and the control group (*p* > 0.05).

### 3.5. Influence of Vitamin D Status at Baseline on the Intervention Outcomes

The number of participants with inadequate and adequate vitamin D status at baseline, after 4 weeks of intervention, and after 8 weeks of intervention compared with the reference value of 50 nmol/L and 75 nmol/L is presented in Table 6. Concerning the lower, namely the 50 nmol/L reference value, at baseline, 70% of all participants (*n* = 69) had 25(OH)D concentrations ≥50 nmol/L, after 4 weeks of intervention 66% (*n* = 65), while after 8 weeks of intervention, it was 54% (*n* = 53). Interestingly, no differences between the intervention groups and the control group were observed (*p* > 0.05). None of the participants’ 25(OH)D serum concentrations exceeded the potentially toxic concentration of 250 nmol/L at any measuring time. Regarding the higher reference value, namely 75 nmol/L, at baseline, only 31% of all participants (*n* = 31) had 25(OH)D serum concentrations ≥75 nmol/L, after 4 weeks of intervention 18% (*n* = 18), while after 8 weeks of intervention, it was 15% (*n* = 15). Just like for the previously analyzed reference value of 50 nmol/L, no differences were observed between the groups (*p* > 0.05).

The comparison of total 25(OH)D serum concentrations before, after 4 weeks, and after 8 weeks of the intervention, as well as its changes throughout the study between subgroups characterized by an adequate (25(OH)D ≥ 50 nmol/L) or inadequate (25(OH)D < 50 nmol/L) status at baseline, is presented in Table 7. What could be anticipated was that there were differences between the 25(OH)D serum concentration at baseline between the participants with an adequate and inadequate vitamin D baseline status in all three study groups (smoked salmon: adequate 69.3 vs. inadequate 39.5 nmol/L; *p* < 0.001; salmon sausage: adequate 79.0 ± 19.8 vs. inadequate 40.7 ± 8.8 nmol/L; *p* < 0.001; control: adequate 76.9 vs. inadequate 36.8 nmol/L; *p* < 0.001). Interestingly, similar differences were observed after 4 and 8 weeks of intervention in all three groups, including the intervention groups, indicating that the intervention was not sufficient to overcome the differences of 25(OH)D serum concentration at baseline. However, the 25(OH)D serum concentration change from w0 to w5 did differ between the subgroups of adequate and inadequate baseline vitamin D status among the participants from the salmon sausage group (−7.2 ± 7.7 vs. 1.5 ± 6.1 nmol/L; *p* = 0.018). In the salmon sausage group, it also differed for the w0 to w9 change (−5.4 vs. −3.0 nmol/L; *p* = 0.048). This indicates that the intervention was more efficacious among participants with an inadequate baseline vitamin D status. No differences were observed in the 25(OH)D serum concentration changes in the control group, while in the smoked salmon group, a non-significant but close-to-statistically-significant difference was noted for the w0 to w5 period (−9.3 vs. −3.8 nmol/L; *p* = 0.079) and the w0 to w9 period (−12.5 vs. −5.8 nmol/L; *p* = 0.055).

### 3.6. Intervention Efficacy

The number of participants whose 25(OH)D serum concentration increased or was maintained compared to those whose 25(OH)D decreased during the different study periods is presented in Table 8. During the first 4 weeks of the intervention (from w0 to w5), an increase/maintenance of 25(OH)D was observed in 17% (*n* = 17) of all participants, during the last 4 weeks of the intervention (from w5 to w9) in 20% (*n* = 20), while during the whole intervention time in 9% (*n* = 9). Interestingly, while from w0 to w5 only a non-significant but close-to-statistically-significant difference was observed between the groups (*p* = 0.097), from w5 to w9, in a pairwise comparison, differences were observed between the salmon sausage and the control group (*p* = 0.015), and a non-significant but close-to-statistically-significant difference was observed between the smoked salmon and the control group (*p* = 0.077), while no difference was noted between the smoked salmon and salmon sausage group (*p* > 0.05). From w0 to w9, in a pairwise comparison, a non-significant but close-to-statistically-significant difference was observed when the salmon sausage and the control group, as well as when the smoked salmon and salmon sausage group, were compared (*p* = 0.052; *p* = 0.095; respectively), while no differences between the smoked salmon group and the control group (*p* > 0.05) were noted.

The analysis of the number of participants whose 25(OH)D serum concentration increased or was maintained compared to those whose 25(OH)D decreased during the different study periods with both intervention groups combined is presented in Supplementary Table S2.

### 3.7. Additional Observations

After the 8-week intervention, three participants (11%) from the salmon sausage group, twelve participants (32%) from the smoked salmon group, and one (3%) from the control group reported improved dermatological health, while two (7%) from the salmon sausage group and three (9%) from the control group reported dermatological deterioration. There were differences between the three groups (*p* = 0.008), as well as between the two intervention groups (*p* = 0.049) with more participants observing dermatological health improvements and less deterioration in the smoked salmon group than in the salmon sausage group. Specifically, in the smoked salmon group, ten participants (29%) reported improved complexion, one (3%) noted enhanced hair condition and one (3%) observed improvement in hair, skin, and nail condition. In the salmon sausage group, two participants (7%) reported an improvement in complexion, one (4%) noted an enhancement in hair and nail condition, and two participants (7%) experienced a deterioration in complexion. In the control group, one (3%) reported improved complexion, two (6%) observed a deterioration in complexion and appearance of acne, and one (3%) noted poorer hair condition.

Most participants from the smoked salmon group declared that they were fond of the taste, texture, and overall quality of the intervention product and 20 participants from this group (53%) would have wanted the study to continue longer. The ones who did not want it to continue were tired of having to consume the same product so frequently, and some were put off by its smell at the end of the 8-week intervention. On the other hand, many said they got used to consuming smoked salmon regularly and would miss it. In the salmon sausage group, seven participants (26%) from the group declared that they would have liked the study to continue longer, while 20 participants (74%) reported being jaded by the taste and texture of the daily consumed product and were pleased that the intervention lasted only 8 weeks.

## 4. Discussion

### 4.1. Serum Concentration Changes of 25(OH)D After 8 Weeks of Intervention

In the previously conducted VISA study [26], a surprising decrease in the 25(OH)D serum concentration was observed despite consuming more salmon, being a source of vitamin D. However, no control group or other intervention groups were included in it; therefore, the present study (VISA 2) provides a more profound insight. To start with, in the present VISA 2 study, a decrease in the 25(OH)D serum concentration was seen in both intervention groups, as well as in the control group. However, what should be underlined is that the decrease was three and a half times greater in the control group than in the salmon sausage intervention group (−15.0 nmol/L vs. −4.3 nmol/L from w0 to w9). This indicates a positive influence of incorporating 100 g of salmon sausage as a source of vitamin D into the daily diet to slow down the natural decline of 25(OH)D due to the lack of cutaneous vitamin D synthesis in Poland in autumn and winter [10].

Surprisingly, no statistically significant difference was observed comparing the smoked salmon group in which the median decrease from w0 to w9 was 9.3 nmol/L, and the control group in which it was 15.0 nmol/L. Moreover, the decrease after 8 weeks of intervention in the smoked salmon group was significantly greater than in the salmon sausage group. This suggests that the daily intake of 25 g of smoked salmon did not have a distinct influence on vitamin D status compared to not consuming it (like in the case of the control group), in contrast to 100 g of salmon sausage (both of which provided around 5 µg of vitamin D daily). Moreover, in the previously conducted VISA study [26] (which started and finished on exactly the same day and month 4 years before to avoid possible differences due to the time of the year), the medium decrease of 25(OH)D after 8 weeks of intervention with smoked salmon was 8.1 nmol/L compared to 9.3 nmol/L in the present VISA 2 study. It seems unexpected as the daily intake of smoked salmon in the VISA study [26] was 50 g daily (providing around 10 µg of vitamin D daily), while in the present VISA 2 study, it was twice as small, so it provided two times less vitamin D per day. The fact that the decline in the groups consuming 25 g and 50 g of smoked salmon per day was so similar (9.3 and 8.1 nmol/L, respectively), as well as the fact that consuming 25 g and being in the control group did not have a different influence on the 25(OH)D decline after 8 weeks of intervention, indicates that there most probably may have been some factors influencing the absorption/metabolism of the vitamin D from smoked salmon. On the other hand, despite the similar, but higher in smoked salmon, amount of vitamin D in the daily portion of smoked salmon and salmon sausages (5.3 vs. 4.4 μg, respectively), a positive influence compared to the control group was seen only in the salmon sausage group, suggests that vitamin D is better absorbed/metabolized from salmon sausages than from smoked salmon. While to our best knowledge such differences have not yet been clearly stated in the literature, some possible explanations for this phenomenon were identified.

### 4.2. Possible Explanations

#### 4.2.1. Fat Content

First of all, what differentiated the products used in the intervention was their fat content. While the daily portion of salmon sausage provided 16.7 g of fat, the daily portion of smoked salmon provided 3.25 g, more than five times less. Since vitamin D is fat-soluble, its absorption could be enhanced when taken with a meal high in fat [44]. In vitro gastrointestinal model studies show that vitamin D bioaccessibility depends on the matrix (food) composition, including its fat content [30]. However, results from human studies regarding the direction of the influence are varied.

On the one hand, a study by Raimundo et al. [44] indicated that when a capsule containing 1250 µg of vitamin D3 was ingested with a high-fat meal (25.6 g of fat in a 473-kcal meal, corresponding to 48.7% of energy from fat), 25(OH)D serum concentrations were higher 7 and 14 days later compared to when the capsule was ingested with a low-fat meal (1.7 g of fat in a 465-kcal meal, corresponding to 3.3% of energy from fat). Another study of a similar design conducted by Raimundo et al. [45] indicated that the increase in 25(OH)D serum concentration 2 weeks after taking a vitamin D supplement was greater when the supplement was consumed with a meal containing at least 15 g of fat compared to a fat-free meal. This aligns well with the presented results, as the fat content of the daily salmon sausage portion was 16.7 g (more than 15 g), and most of the participants consumed it during one meal. Moreover, a study by Dawson-Hughes et al. [46] showed that plasma cholecalciferol was higher when participants took a 1250 µg vitamin D supplement with a meal containing fat than a fat-free meal, whereas the 25(OH)D serum concentration was not assessed. On the other hand, the results from a different intervention study by Dawson-Hues et al. [47] indicated that while 12 h after receiving 1250 µg of vitamin D3, the plasma cholecalciferol increases were highest in the low-fat meal group compared to the high-fat and no-meal group, the 25(OH)D increases 30 and 90 days later did not differ between the groups.

Despite observing no rise in the present study, the results seem to correspond to the results from Raimundo et al. [44,45], as the 25(OH)D decline was smaller in the intervention group consuming salmon sausages which contained more fat than smoked salmon. What connects these studies to the present study is the young age of the participants (below 30 years) in contrast to the older participants in the study by Dawson-Hues et al. [47] (mean age 58 years), where no difference between the groups was observed. This might suggest that the intake of vitamin D3 (in the form of a supplement or a naturally-rich-in-vitamin-D product such as salmon sausage) results in higher 25(OH)D levels if ingested with more fat only in young individuals. Whether a smaller decline (like in the present study) or a higher increase (like in the Raimundo et al. study [44]) will be observed will probably depend on the amount of vitamin D3 ingested. While in the Raimundo et al. [44] study it was 1250 µg at one time, in the present study, it was 5 µg daily, corresponding to 280 µg during the whole 8-week-long intervention.

What should not be overlooked, however, is that in the cited studies [44,46,47], a high dose of a supplement was given to their participants in contrast to the present study in which the participants received much smaller amounts of vitamin D in the form of a food product. This might result in a markedly different vitamin D uptake mechanism, as the findings of Reboul et al. [48] indicate that at low dietary doses, the absorption of vitamin D is primarily protein-mediated. In contrast, at high pharmacological doses, passive diffusion probably occurs [48].

Although in the present study, the higher fat content of salmon sausages seemed to improve the effect of the intervention, other clinical studies indicate that vitamin D can also be effectively absorbed from food products fortified with vitamin D, even if low in fat, such as orange juice [49], low-fat cheese [50], and bread [51], suggesting that 25(OH)D increases can be observed even if the intervention products do not contain high amounts of fat [49,50,51] contrary to the hypothesis described above.

However, it should be mentioned that one more explanation connected with the fat content of the products might be the different types of fatty acids found in them. They may have an effect, as Holmberg et al. [52] indicated that vitamin D3 supplements increase cholecalciferol serum concentrations more effectively when vitamin D3 is administered in fats containing long-chain triglycerides compared to medium-chain triglycerides, suggesting that the presence of long-chain fatty acids facilitates vitamin D3 absorption [52]. Moreover, a diet rich in monounsaturated fatty acids (MUFA) was found to be more effective in increasing cholecalciferol concentrations compared to a diet rich in polyunsaturated fatty acids (PUFA), as the latter seems to reduce the effectiveness of vitamin D3 supplementation [53]. However, the study by Holmberg et al. [52] also showed that when the vitamin D3 supplement was administered with food, the difference between the two formulas was not observed, while the 25(OH)D3 differences were non-significant and did not allow for any specific conclusions. Moreover, a different trial indicated that the PUFA and MUFA ratios do not influence vitamin D3 absorption [46]. Hence, it seems that to date it still is an unsolved problem whether or not the different fatty acids influence vitamin D absorption.

#### 4.2.2. Production Process

Secondly, the surprising results could be explained by the food matrix, particularly considering that salmon sausages are highly processed food products that have undergone homogenization. The matrix may play a role as it is believed that vitamin D must be released from its food or supplement matrix to become bioaccessible, meaning it needs to be solubilized in micelles to be available for absorption [54]. However, clinical studies indicate that the matrix does not seem to influence the bioavailability of vitamin D when the matrix is bread [51], mushrooms [55], or cheese [50], compared to a supplement. Nevertheless, to the best of our knowledge, studies on the bioavailability of vitamin D from fish-based sausages have not yet been conducted. Additionally, unlike the mentioned studies [50,51,55] that compared the bioavailability of vitamin D from food products with its bioavailability from a supplement, the present study compared two different food products rather than a supplement. Interestingly, in the mushroom bioavailability study [55], lyophilized and homogenized mushrooms were used, while the bioavailability of vitamin D from fresh nonlyophilized mushrooms was not conclusive, which might suggest that the process of lyophilization and homogenization was essential concerning vitamin D bioavailability from the mushrooms. This corresponds to the fact that in the present intervention salmon sausages, which are homogenized during the production, were far more efficacious than smoked salmon.

Not only may the homogenization of salmon sausages have influenced vitamin D availability, but also the applied thermal treatment may have had an impact. A study assessing the impact of various cooking methods of pork products on vitamin D content showed that the vitamin D3 concentration was highest in raw and cooked sausage compared to minced meat and loin, as well as that the vitamin D activity was also highest in cooked sausage [56]. What is more, vitamin D3 concentrations as well as vitamin D activity increased for all cooking treatments, probably owing to water/fat loss. However, in the present study, while vitamin D3 concentrations (µg/g) were not the same in smoked salmon and salmon sausage, the daily portion of the product was of such a size to provide a similar (around 5 µg) vitamin D3 amount. Whether or not cooking, which was allowed in the present study, could have impacted the results also seems unclear, as some studies show that vitamin D retention after cooking is higher than in raw products [56], while others show that the retention decreases after cooking treatments [57].

Importantly, when vitamin D3 content in food is assessed, the analyzed samples are usually homogenized [51,55,56,57], which is also true in the case of fish [58]. Therefore, the much better results from consuming salmon sausage compared to smoked salmon could be explained by the fact that the assessed vitamin D content in smoked salmon may not reflect the amount of vitamin D that is bioavailable for humans, as the product is not homogenized before eating. Not everyone bites and chews thoroughly enough to ensure that the product is fully homogenized before swallowing. In contrast, salmon sausages are already homogenized before consumption. It is known that vitamin D3 can only be absorbed when it is dissolved in small enough particulates that can pass the mucus layer [59]. Also, it is hypothesized that protein-digesting enzymes such as pepsin and trypsin play a crucial role in vitamin D absorption by breaking down vitamin D binding proteins found in food, thereby aiding in its release. In the duodenum, however, digestive enzymes such as amylases, lipase, and protease continue to facilitate the release of vitamin D from the food matrix [60].

Homogenized products offer a greater surface area for digestive enzymes to work compared to non-homogenized ones. That is why, despite no rigid proof, it is probable that the homogenization process being part of salmon sausage production might be a factor in improving vitamin D bioavailability and hence in improving 25(OH)D in blood, as the actual available amount of vitamin D3 is higher from salmon sausages than from smoked salmon despite what laboratory analyses indicate. However, no studies concerning the effect of product homogenization on vitamin D bioavailability have been conducted so far; therefore, it is only a hypothesis. What is stated by other scientists, however, is that there is limited research on how the complexity of the food matrix affects the absorption and bioavailability of vitamin D and that more studies are needed to better understand it [60].

#### 4.2.3. Frequency of Consuming the Intervention Product

Participants were allowed to either eat the daily portion of the intervention product every day or to eat twice the daily portion every second day. It was noted that participants from the salmon sausage group consumed the intervention product more often on a daily basis compared to participants from the smoked salmon group. This might help explain the observed results, as the actual dose of vitamin D was more often 5 µg daily in the salmon sausage group, while it was typically 10 µg every second day in the smoked salmon group. There are signals that the conversion rate to 25(OH)D may be slower in subjects receiving large doses of vitamin D [61], which, with the above-mentioned analysis, might suggest that providing the body with smaller amounts of vitamin D but more frequently may be more effective in improving 25(OH)D status than bigger amounts but less frequently. This could help clarify the results from the present study, as salmon sausages, which were consumed more often daily, therefore providing smaller amounts of vitamin D but more frequently, were found to be more efficient than smoked salmon, which was more often consumed every second day, providing larger amounts of vitamin D but less frequently.

#### 4.2.4. Dietary Habits Before Intervention

Analyses concerning fish intake among the participants indicated that while the weekly intake of fish and fish products at baseline (before the intervention) did not differ between the groups, more participants from the salmon sausage group complied with the recommendation to consume at least 150 g of fish and fish products weekly than participants from the smoked salmon and the control group. This and the fact that the intervention was far more effective among participants from the salmon sausage group compared to those from the smoked salmon group might indicate that consuming at least 150 g of fish weekly before the intervention might have somehow improved vitamin D absorption and bioavailability during the intervention. To the best of our knowledge, the concept that one’s gut/body might adapt to learn how to digest food products or absorb/metabolize vitamin D has not been described in literature yet. However, there are indications that some digestive enzymes adapt to the diet [62]. Therefore, some enzymes involved in the digestion of salmon may also be adaptive. If that is the case, the activity of these enzymes might be higher among people who habitually comply with fish intake recommendations (like in the case of the participants from the salmon sausage group).

Similarly, when analyzing the number of participants whose 25(OH)D serum concentration increased or was maintained compared to those whose levels decreased during different study periods, the *p*-value indicates that while only a non-significant but close-to-statistically-significant difference was observed during the first 4 weeks of the intervention, a significant difference emerged during the final 4 weeks (from w5 to w9). Specifically, a larger proportion of individuals in the salmon sausage group showed an increase or maintenance of 25(OH)D levels compared to the control group, with a similar non-significant but close-to-statistically-significant difference noted when comparing the smoked salmon group to the control group. This might be related to the time of the year when the study was conducted, namely autumn, a period during which vitamin D is no longer synthesized in the skin in Poland [10] and must be obtained through diet or stored reserves. However, to the best of our knowledge, little is known about specific physiological processes occurring in the human body during this time.

#### 4.2.5. Physiological Explanation Connected to Vitamin D Metabolism in the Body

Upon entry into the human body, either through cutaneous synthesis or dietary intake, vitamin D binds to vitamin D-binding protein (VDBP), enabling its transport through the circulatory system, either directly to hepatic tissue or to be stored in adipocytes [63]. The liver is the primary site for the first phase of vitamin D metabolism, namely 25-hydroxylation, which converts vitamin D to 25-hydroxyvitamin D (25(OH)D), the form quantified in the present study [64]. Although recent findings suggest other tissues may contribute to this process, the enzyme CYP2R1 remains the principal 25-hydroxylase in the hepatic metabolism of vitamin D [65]. Subsequently, 25(OH)D is metabolized by the enzyme CYP27B1 to 1,25-dihydroxyvitamin D (1,25(OH)_2_D), which occurs principally in the kidney. This form is responsible for the majority of vitamin D hormonal activity and biological actions [65]. The concentration of 1,25(OH)_2_D is tightly regulated by CYP24A1, a 24-hydroxylase enzyme, which is up-regulated by 1,25(OH)_2_D to promote its own catabolism [65], as well as the catabolism of 25(OH)D [64].

The renal production of 1,25(OH)_2_D is known to be tightly regulated and inhibited by factors such as calcium and phosphate [65]. Even a slight decrease in extracellular fluid calcium, for instance, after an overnight fast, can trigger a cascade of biological processes that may also affect vitamin D metabolism [66]. Furthermore, since magnesium plays a critical role in the transport of vitamin D and the hydroxylation steps required to produce both 25(OH)D and 1,25(OH)_2_D, as it functions as a cofactor, magnesium deficiency may impair vitamin D activation [67].

Therefore, it can be hypothesized that the proportion of vitamin D transported to the liver and subsequently converted to 25(OH)D, detectable in blood tests, may have been influenced by other hepatic metabolic pathways modulated by various food-derived nutrients. Additionally, the participants’ 25(OH)D concentration could have been affected by the concentrations of 1,25(OH)_2_D, which were not measured in the current study. Furthermore, some of the vitamin D absorbed from the salmon products may have been directly transported to and stored in adipose tissue, rather than serving as a substrate for 25(OH)D hydroxylation in the liver, and thus not detected in blood tests. According to Heaney et al. [68], vitamin D stored in adipose tissue is estimated to account for approximately 17% of the administered dose, with significant individual variation, which underscores the importance of considering individual differences.

### 4.3. Baseline 25(OH)D Serum Concentration as an Important Factor for the Intervention Efficacy

The results from the present study indicate that the baseline 25(OH)D is a factor that should be taken into consideration when assessing the efficacy of a vitamin D-focused dietary intervention. In the salmon sausage group, where the intervention was more efficacious compared to the smoked salmon group, a mean increase in 25(OH)D serum concentration was observed after 4 weeks among participants with a baseline 25(OH)D serum concentration below 50 nmol/L (indicative of inadequate vitamin D status). In contrast, a decrease was noted among those with baseline 25(OH)D serum concentrations of 50 nmol/L or higher (adequate vitamin D status). Over the whole intervention period (from week 0 to week 9), decreases were observed in the salmon sausage group regardless of the baseline 25(OH)D status. However, the decreases were more pronounced among participants with an adequate baseline vitamin D status compared to those with an inadequate baseline vitamin D status.

In the smoked salmon group, a non-significant but close-to-statistically-significant difference was observed concerning the change in 25(OH)D serum concentration from w0 to w9 depending on the baseline vitamin D status. The median decrease among participants with an adequate baseline vitamin D status was greater than among those with an inadequate vitamin D status at baseline. In the control group, the decreases in 25(OH)D serum concentration were similar across both baseline vitamin D status groups. These findings suggest that dietary interventions aiming at increasing vitamin D intake, like the one in this study, are more effective among individuals with inadequate vitamin D status. Therefore, the recommendation to consume more vitamin D-rich fish may be especially important and beneficial for individuals with low 25(OH)D serum concentrations.

Moreover, another benefit related to increasing vitamin D-rich fish intake might also be the improvement of dermatological health, such as the condition of complexion, hair, and nails, which were reported by some of the study participants despite not being the aim of the present study. These observations can be explained by other studies indicating the essential beneficial role of omega-3 fatty acids and vitamin D, both present in salmon [69], in dermatological conditions [70,71].

Other studies also confirm that interventions induce greater changes in 25(OH)D levels when starting from low baseline concentrations compared to high baseline levels [72]. In the previously cited single-blind bioavailability study by Natri et al. [51], a negative correlation was observed between the initial 25(OH)D serum concentration and the increase in 25(OH)D serum concentration, indicating that supplementation and food fortification were more effective in individuals with initial low vitamin D levels. Additionally, some studies on moderate vitamin D supplementation are designed to include only participants with low baseline 25(OH)D levels to ensure the experiment demonstrates a measurable difference [55].

However, individual differences cannot be forgotten. In the current study, there were participants in both intervention groups whose 25(OH)D levels increased after 4 and 8 weeks of the intervention despite having adequate baseline vitamin D status, as well as participants whose 25(OH)D levels decreased despite having inadequate baseline vitamin D status.

### 4.4. Strengths and Limitations of the Study

The major strength of the study is its evaluation of the impact of adhering to fish consumption recommendations on vitamin D status in autumn within a real-life context, unlike many studies that assess such impacts using unrealistically large fish portions, which are much higher than the minimal recommended amounts [27]. Additionally, the study included participants with both adequate and inadequate baseline 25(OH)D concentrations, reflecting typical societal variations. Furthermore, it is the first study to examine the influence of salmon sausage intake on vitamin D status and the first to compare the effects of increasing the consumption of the same fish species, namely salmon, in two different forms. Notably, 25(OH)D decreases were observed not only in the control group but also in the intervention group. Despite similar vitamin D intakes and no cutaneous vitamin D synthesis, the study found that the efficacy of the intervention was significantly higher with salmon sausage compared to smoked salmon. These findings are unique and contribute valuable insights.

Despite the novel observations from the conducted study, some limitations should be noted. First, participants were permitted to consume the intervention products either daily, also in multiple portions, or every other day, and at any meal they preferred. Given the significant differences in the intervention efficacy between the two groups, it might have been beneficial to impose more rigid consumption rules to better understand the causes of these differences. However, the decision to allow more flexibility was made to assess the impact of increasing fish intake to recommended levels on vitamin D status in a real-life context. Additionally, because vitamin D is fat-soluble [45] and stored in fat tissue, it is believed by some authors that daily consumption is not necessary [73]. Moreover, the flexible consumption rules likely enhanced intervention compliance, as reported by the participants.

Another limitation is the relatively short intervention period of 8 weeks. Given the surprising results, a longer intervention might have provided a deeper insight into the effect of increased vitamin D-rich fish consumption on 25(OH)D levels in autumn. However, a meta-analysis indicated that even a 4-week intervention is sufficient to observe differences in vitamin D status [27]. Also, it seems that longer-lasting interventions should be planned differently, as three-quarters of the participants from the salmon sausage group expressed feeling bored with the taste and texture of the daily consumed product and were relieved that the intervention lasted only 8 weeks. Additionally, the drop-out rate due to non-adherence to the intervention from this group was much higher than from the smoked salmon and the control group.

What is more, since fat intake seems to influence vitamin D bioavailability or absorption [45], it would have been beneficial to assess the participants’ fat intake, including different fatty acids, both in the meal containing the intervention product and throughout the day. However, a precise analysis of fat intake is challenging, as fat is often consumed unconsciously due to the addition of fat during meal preparation. Last but not least, due to indications that high physical activity may result in lower 25(OH)D concentrations despite higher vitamin D intakes [74], possibly due to 1,25(OH)_2_D utilization in muscle recovery [75], it might have been beneficial to assess the participants’ physical activity. However, it seems that these differences concern males and not females [74]; therefore, since the participants in the present study were females, physical activity has probably not been a distinguishing factor concerning the efficacy of the intervention.

### 4.5. Recommendations for Maintaining Adequate Vitamin D Status

Interestingly, some guidelines such as the Polish ones [17] recommend vitamin D supplements for all age groups and during the whole year and do not highlight the importance of the main and natural sources of vitamin D, namely skin synthesis and food products. To the best of our knowledge, they are the only recommendations that seem to ignore the natural sources of vitamin D. However, others such as the French, the Dutch, the German–Austrian–Swiss (DACH), and the Finnish ones primarily underline the skin synthesis and the diet as sources of vitamin D [76,77,78,79] and state that vitamin D supplementation should be considered when diet does not provide sufficient amounts of vitamin D [79] or when skin synthesis is lacking [78]. The Finnish underline that unnecessary vitamin D supplementation should be avoided [79], while the DACH recommendations indicate that healthy adults with frequent sun exposure may achieve the recommended vitamin D status without the use of vitamin D supplements [78], and the French that through sun exposure and food ensuring an adequate vitamin D status is possible [76]. Because vitamin D is more bioaccessible from food products than from supplements [30]—often requiring much higher supplement doses than the recommended dietary intakes—and because fatty fish provide not only vitamin D but also essential and often deficient omega-3 fatty acids [69], it does seem more beneficial to recommend increasing fatty fish consumption rather than immediately turning to vitamin D supplements.

However, in view of the findings of the current VISA 2 study, it seems that to maintain adequate vitamin D status when sunlight is insufficient for cutaneous vitamin D synthesis, the fatty fish intake must be higher than 175 g weekly.

### 4.6. Proposed Directions for Future Research

In light of the unexpected results that the intervention was more effective when salmon sausages were consumed compared to smoked salmon, and considering the mixed results of research on fish intake and vitamin D status [27], further investigation is needed to determine optimal fish species, quantities, and forms for maintaining adequate 25(OH)D levels in regions with insufficient sunlight for cutaneous vitamin D synthesis during autumn and winter [10,15].

Moreover, given that a significant portion of fish on the market is farmed, with farmed salmon representing 80% of the global supply [80], and considering that wild fish usually have a higher vitamin D content compared to farmed fish [32], as well as higher concentrations of EPA and DHA despite lower fat content [81], the origin of the fish should also be taken into account in future studies. Additionally, with many fish stocks being fully fished or overfished, placing substantial pressure on wild populations [82], and acknowledging that mushrooms can also be a good source of vitamin D [55], as well as the growing popularity of plant-based diets [83], research into the vitamin D content of commercially available mushrooms should also be explored.

## 5. Conclusions

Despite increasing salmon intake to the recommended levels of weekly fish consumption and consequently raising vitamin D intake, vitamin D status could not be maintained during the autumn 8-week dietary intervention among young women. Serum concentrations of 25(OH)D decreased in both of the two intervention groups. However, the decrease in 25(OH)D was significantly smaller in the salmon sausage group compared to the smoked salmon and the control group. Surprisingly, the decrease after the intervention did not differ between the smoked salmon and the control group. These results suggest that the intervention was far more effective when salmon sausages were consumed rather than smoked salmon, despite both providing similar quantities of vitamin D and a higher total vitamin D intake in the smoked salmon group. Possible explanations include the higher fat content in salmon sausages compared to smoked salmon, the fact that salmon sausages were homogenized while smoked salmon was not, as well as other factors that may have influenced the metabolism of 25(OH)D.

Also, smaller decreases in 25(OH)D were observed among participants with an inadequate baseline vitamin D status compared to those with an adequate vitamin D status. Therefore, it might be concluded that incorporating salmon sausages into the daily diet may aid slow down the natural decline of 25(OH)D in autumn in young women, especially those with very low 25(OH)D concentrations. However, the study also indicates that individual differences cannot be forgotten. Further research is needed to explain the observed differences as it seems that there might be other, not-yet-fully-understood factors, which influence vitamin D absorption and/or metabolism.

## Figures and Tables

**Table 1:** Nutritional value of the smoked salmon and salmon sausages used in the intervention (presented per 100 g, corresponding to the daily serving of salmon sausage and four daily servings of smoked salmon planned within the dietary intervention).

	Smoked Salmon, 100 g	Salmon Sausage, 100 g
Energy, kcal	176.0	241.0
Fat, g	9.9	16.7
Saturated fatty acids, g	1.7	2.6
Carbohydrates, g	1.0	11.4
Protein, g	20.1	11.6
Salt, g	1.7	1.5
Omega-3 fatty acids, g	1.4	-
EPA + DHA, mg	610	360
Vitamin D3, μg	21.3 ± 5.55	4.41 ± 1.15

EPA + DHA—the sum of eicosapentaenoic (EPA) and docosahexaenoic (DHA) acid.

**Table 2:** Anthropometric characteristics of the two intervention groups and the control group at baseline.

	Smoked Salmon Intervention Group *n* = 38		Salmon Sausage Intervention Group *n* = 27		Control Group *n* = 34		p **
	Mean ± SD	Median (P25; P75)	Mean ± SD	Median (P25; P75)	Mean ± SD	Median (P25; P75)	
Age, years	21.6 ± 2.7	21 (20; 23) *^a^	22.4 ± 2.8	21 (20; 23.5) *^ab^	23.2 ± 2.9	23 (21; 25) ^b^	0.048
Weight, kg	58.3 ± 7.2	58.5 (51.8; 62.8)	63.0 ± 9.6	61.5 (55.4; 69.4) *	59.8 ± 8.9	60.3 (52.7; 65.9)	0.172
Height, cm	168.1 ± 7.0	167.8 (163.7; 174.1)	168.4 ± 6.5	168.2 (164.2; 171.8)	168.3 ± 6.4	167.6 (164.5; 171.8)	0.982
BMI, kg/m^2^	20.6 ± 2.4	20.4 (19.2; 22.5)	22.2 ± 3.0	21.2 (20.1; 23.7) *	21.1 ± 3.0	20.9 (18.8; 22.8)	0.118
SLM, %	70.8 ± 5.1	70.9 (66.8; 74.1)	67.4 ± 4.9	67.3 (65.0; 69.9)	68.9 ± 5.6	69.3 (64.9; 72.4)	0.299
TBW, %	55.9 ± 4.0	56.0 (52.6; 58.4)	53.1 ± 3.8	53.0 (51.1; 55.0)	54.3 ± 4.4	54.6 (51.2; 57.1)	0.291
FM, %	23.7 ± 5.5	23.7 (20.3; 28.0) ^a^	27.4 ± 5.2	27.5 (24.8; 30.0) ^b^	25.8 ± 6.0	25.2 (22.0; 30.0) ^ab^	0.014
FFM, %	76.3 ± 5.5	76.3 (72.0; 79.7)	72.6 ± 5.2	72.5 (70.0; 75.2)	74.2 ± 6.0	74.8 (70.0; 78.0)	0.302
SMM, %	42.5 ± 3.1	42.5 (40.1; 44.4)	40.4 ± 2.9	40.3 (38.9; 41.9)	41.3 ± 3.4	41.6 (39.0; 43.4)	0.298

* non-normal distribution (verified using Shapiro–Wilk test; *p* ≤ 0.05); ** analysis of variance (ANOVA) test (for normal distribution) or Kruskall–Wallis ANOVA test (for non-normal distribution); different letters in rows (a, b) indicate significant differences between groups (*p* < 0.05)—Tukey’s post hoc analysis; SLM—Soft lean mass; TBW—Total body water; FM—Fat mass; FFM—Fat-free mass; SMM—Skeletal muscle mass.

**Table 3:** Comparison of the weekly intake of fish and fish products among the participants from the different study groups at baseline, during the 4 first weeks and the last 4 weeks of the intervention.

Weekly Intake of Fish and Fish Products, g/week		Smoked Salmon Intervention Group *n* = 38		Salmon Sausage Intervention Group *n* = 27		Control Group *n* = 34		p **
		Mean ± SD	Median (P25; P75)	Mean ± SD	Median (P25; P75)	Mean ± SD	Median (P25; P75)	
w0 (baseline)		99.7 ± 58.7	87.5 (56.1; 137.0) *	131.0 ± 75.5	128.0 (75.8; 187.0)	108.0 ± 105.0	78.8 (54.0; 128.0) *	0.119
w1 to w4 (4 first weeks of intervention)	including intervention	240.0 ± 72.1	222.0 (187.0; 254.0) *^a^	735.0 ± 85.7	712.0 (671.0; 776.0) *^c^	82.9 ± 75.4	64.2 (24.8; 114.0) *^b^	<0.001
	excluding intervention	76.8 ± 72.1	58.3 (23.3; 90.4) *	79.5 ± 79.6	58.3 (17.5; 123.0) *	82.9 ± 75.4	64.2 (24.8; 114.0) *	0.902
w5 to w8 (4 last weeks of intervention)	including intervention	229.0 ± 72.8	210.0 (171.0; 271.0) *^a^	711.0 ± 58.2 ^c^	700.0 (665.0; 747.0)	69.7 ± 57.1	58.3 (26.3; 102.0) *^b^	<0.001
	excluding intervention	66.2 ± 72.7	46.7 (7.3; 108.0) *	59.6 ± 55.3	46.7 (17.5; 93.3) *	69.7 ± 57.1	58.3 (26.3; 102.0) *	0.712

* non-normal distribution (verified using Shapiro–Wilk test; *p* ≤ 0.05); ** Kruskall–Wallis ANOVA test; different letters in rows (a, b, c) indicate significant differences between groups (*p* < 0.05).

**Table 4:** Comparison of the daily intake of vitamin D among the participants from the different study groups at baseline, during the 4 first weeks and the last 4 weeks of the intervention.

Daily Intake of Vitamin D, µg/day		Smoked Salmon Intervention Group *n* = 38		Salmon Sausage Intervention Group *n* = 27		Control Group *n* = 34		p **
		Mean ± SD	Median (P25; P75)	Mean ± SD	Median (P25; P75)	Mean ± SD	Median (P25; P75)	
w0 (baseline)		3.4 ± 1.5	3.1 (2.4; 4.0) *	3.7 ± 1.7	3.4 (2.3; 5.0)	3.0 ± 1.2	2.7 (2.1; 3.4) *	0.152
w1 to w4 (4 first weeks of intervention)	including intervention	7.7 ± 1.2	7.3 (6.8; 8.4) *^a^	7.1 ± 1.8	6.8 (6.0; 7.4) *^b^	2.5 ± 1.1	2.3 (1.8; 3.1) ^c^	<0.001
	excluding intervention	2.7 ± 1.2	2.4 (1.9; 3.4) *	2.9 ± 1.7	2.7 (1.9; 3.2) *	2.5 ± 1.1	2.3 (1.8; 3.1)	0.690
w5 to w8 (4 last weeks of intervention)	including intervention	7.8 ± 1.9	7.3 (6.6; 8.2) *^a^	6.7 ± 1.4	6.5 (5.9; 7.0) *^b^	2.5 ± 1.3	2.3 (1.6; 2.8) *^c^	<0.001
	excluding intervention	2.8 ± 1.9	2.3 (1.7; 3.2) *	2.6 ± 1.4	2.4 (1.8; 2.9) *	2.5 ± 1.3	2.3 (1.6; 2.8) *	0.898

* non-normal distribution (verified using Shapiro–Wilk test; *p* ≤ 0.05); ** Kruskall–Wallis ANOVA test; different letters in rows (a, b, c) indicate significant differences between groups (*p* < 0.05).

**Table 5:** Participants’ total 25(OH)D serum concentrations and their changes at baseline, after 4 weeks, and after 8 weeks of the intervention.

Variables	Time	Smoked Salmon Intervention Group *n* = 38		Salmon Sausage Intervention Group *n* = 27		Control Group *n* = 34		p **
		Mean ± SD	Median (P25; P75)	Mean ± SD	Median (P25; P75)	Mean ± SD	Median (P25; P75)	
25(OH)D serum concentration, nmol/L	w0	64.2 ± 23.2	63.5 (47.8; 77.2)	66.3 ± 24.9	65.8 (48.6; 80.4)	65.1 ± 27.3	61.5 (47.9; 76.4)	0.947
	w5	55.9 ± 18.6	56.9 (41.0; 68.9)	61.9 ± 22.6	57.5 (46.8; 75.5)	53.8 ± 23.4	56.4 (33.9; 69.8)	0.323
	w9	52.6 ± 18.8	53.3 (36.7; 63.0)	62.4 ± 26.5	56.3 (45.8; 68.3) *	48.3 ± 21.8	48.4 (32.6; 62.5)	0.130
25(OH)D serum concentration change, nmol/L	w0 to w5	−8.2 ± 11.6	−6.3 (−12.2; −2.1) *^ab^	−4.3 ± 8.2	−2.5 (−11.4; 0.0) ^b^	−11.4 ± 10.5	−10.0 (−16.1; −4.5) ^a^	0.022
	w5 to w8	−3.3 ± 6.8	−3.1 (−6.1; −0.5) *^ab^	0.4 ± 8.6	−2.3 (−4.5; 3.8) *^b^	−5.5 ± 4.6	−4.4 (−8.4; −2.3) ^a^	0.020
	w0 to w9	−11.6 ± 14.1	−9.3 (−17.6; −3.9) *^a^	−3.9 ± 13.2	−4.3 (−8.5; −0.8) *^b^	−16.9 ± 12.4	−15.0 (−22.0; −8.4) *^a^	<0.001

* non-normal distribution (verified using Shapiro–Wilk test; *p* ≤ 0.05); ** ANOVA Fisher’s test (for normal distribution) or Kruskall–Wallis ANOVA test (for non-normal distribution); 25(OH)D—25-hydroxyvitamin D; different letters in rows (a, b) indicate significant differences between groups (*p* < 0.05); w0—baseline; w5—after 4 weeks of intervention, in week 5; w9—after 8 weeks of intervention, in week 9.

**Table 6:** Number of participants with inadequate and adequate vitamin D status at baseline, after 4 weeks of intervention, and after 8 weeks of intervention, compared with the reference value of 50 nmol/L and 75 nmol/L.

Reference Value	Measurement Time	Vitamin D Status	Smoked Salmon Intervention Group *n* = 38	Salmon sausage Intervention Group *n* = 27	Control Group *n* = 34	*p* *
50 nmol/L ^1^	w0	Adequate	27 (71%)	18 (67%)	24 (71%)	0.922
		Inadequate	11 (29%)	9 (33%)	10 (29%)	
	w5	Adequate	25 (66%)	19 (70%)	21 (62%)	0.781
		Inadequate	13 (34%)	8 (30%)	13 (38%)	
	w9	Adequate	21 (55%)	16 (59%)	16 (47%)	0.614
		Inadequate	17 (45%)	11 (41%)	18 (53%)	
75 nmol/L ^2^	w0	Adequate	12 (32%)	9 (33%)	10 (29%)	0.946
		Inadequate	26 (68%)	18 (67%)	24 (71%)	
	w5	Adequate	6 (16%)	7 (26%)	5 (15%)	0.470
		Inadequate	32 (84%)	20 (74%)	29 (85%)	
	w9	Adequate	6 (16%)	6 (22%)	3 (9%)	0.346
		Inadequate	32 (84%)	21 (78%)	31 (91%)	

^1^ Adequate vitamin D status defined as 25(OH)D ≥ 50 nmol/L, inadequate vitamin D status defined as 25(OH)D < 50 nmol/L; ^2^ Adequate vitamin D status defined as 25(OH)D ≥ 75 nmol/L, inadequate vitamin D status defined as 25(OH)D < 75 nmol/L; * chi^2^ test; w0—baseline; w5—after 4 weeks of intervention, in week 5; w9—after 8 weeks of intervention, in week 9.

**Table 7:** Comparison of total 25(OH)D serum concentrations before, after 4 weeks, and after 8 weeks of the intervention between subgroups characterized by an adequate (25(OH)D ≥ 50 nmol/L) or inadequate (25(OH)D < 50 nmol/L) status at baseline within the studied groups.

Variables	Time	Vitamin D Status at Baseline	Smoked Salmon Intervention Group (Adequate ^1^ at Baseline: *n* = 27, Inadequate ^2^ at Baseline: *n* = 11)			Salmon Sausage Intervention Group (Adequate ^1^ at Baseline: *n* = 18, Inadequate ^2^ at Baseline: *n* = 9)			Control Group (Adequate ^1^ at Baseline: *n* = 24, Inadequate ^2^ at Baseline: *n* = 10)		
			Mean ± SD	Median (P25; P75)	p **	Mean ± SD	Median (P25; P75)	p **	Mean ± SD	Median (P25; P75)	p **
25(OH)D serum concentration, nmol/L	w0	Adequate ^1^	74.6 ± 18.7	69.3 (62.5; 83.6) *	<0.001	79.0 ± 19.8	74.6 (66.0; 87.5)	<0.001	76.9 ± 22.8	71.5 (60.2; 84.7) *	<0.001
		Inadequate ^2^	38.5 ± 7.8	39.5 (34.0; 44.1)		40.7 ± 8.8	41.8 (34.3; 47.5)		36.8 ± 11.6	39.6 (32.4; 45.8)	
	w5	Adequate ^1^	63.8 ± 13.8	62.3 (54.9; 74.4)	<0.001	71.8 ± 20.6	67.3 (58.4; 79.8) *	<0.001	64.4 ± 18.5	61.3 (54.8; 72.9)	<0.001
		Inadequate ^2^	36.7 ± 14.6	36.0 (25.8; 39.0) *		42.2 ± 10.3	46.0 (38.8; 47.3) *		28.1 ± 9.9	27.9 (21.6; 32.3)	
	w9	Adequate ^1^	59.8 ± 13.9	59.5 (50.0; 68.9)	<0.001	72.0 ± 26.9	63.8 (56.2; 84.6) *	<0.001	57.9 ± 18.0	56.1 (47.2; 67.8)	<0.001
		Inadequate ^2^	34.9 ± 17.9	28.0 (26.8; 35.5) *		43.1 ± 10.4	45.3 (44.5; 46.5) *		25.1 ± 9.0	24.6 (19.1; 30.9)	
25(OH)D serum concentration change, nmol/L	w0 to w5	Adequate ^1^	−10.8 ± 11.6	−9.3 (−14.1; −3.6) *	0.079	−7.2 ± 7.7	−3.8 (−13.1; −1.1)	0.018	−12.5 ± 12.0	−12.0 (−17.9; −2.2)	0.558
		Inadequate ^2^	−1.8 ± 9.3	−3.8 (−7.3; 0.0)		1.5 ± 6.1	0.0 (−2.5; 3.8)		−8.7 ± 5.0	−8.0 (−11.8; −4.6)	
	w5 to w9	Adequate ^1^	−3.9 ± 5.0	−3.3 (−6.8; −1.3)	0.551	0.2 ± 10.0	−3.5 (−4.9; 4.8) *	0.217	−6.6 ± 5.0	−6.0 (−10.2; −2.8)	0.576
		Inadequate ^2^	−1.8 ± 10.0	−2.0 (−5.1; 0.1)		0.8 ± 5.0	−1.5 (−2.3; 1.5) *		−3.0 ± 1.8	−3.5 (−4.4; −2.3)	
	w0 to w9	Adequate ^1^	−14.8 ± 12.9	−12.5 (−21.0; −5.3) *	0.055	−7.0 ± 13.9	−5.4 (−16.3; −1.6)	0.048	−19.0 ± 13.9	−18.5 (−24.0; −8.0)	0.117
		Inadequate ^2^	−3.6 ± 14.3	−5.8 (−11.0; −3.0) *		2.3 ± 9.7	−3.0 (−4.3; 7.8) *		−11.6 ± 4.8	−12.8 (−14.6; −9.0)	

^1^ Adequate baseline vitamin D status defined as 25(OH)D ≥ 50 nmol/L; ^2^ inadequate baseline vitamin D status defined as 25(OH)D < 50 nmol/L; * non-normal distribution (verified using Shapiro–Wilk test; *p* ≤ 0.05); ** U Mann–Whitney test (comparison inside one group); 25(OH)D—25-hydroxyvitamin D; w0—baseline; w5—after 4 weeks of intervention, in week 5; w9—after 8 weeks of intervention, in week 9.

**Table 8:** The number of participants whose 25(OH)D serum concentration increased or was maintained compared to those whose 25(OH)D decreased during the different study periods.

Time	25(OH)D Serum Concentration Change	Smoked Salmon Intervention Group *n* = 38	Salmon Sausage Intervention Group *n* = 27	Control Group *n* = 34	*p* *
w0 to w5	Increase/maintained	6 (16%)	8 (30%)	3 (9%)	0.097
	Decrease	32 (84%)	19 (70%)	31 (91%)	
w5 to w9	Increase/maintained	9 (24%)	9 (33%)	2 (6%)	0.024
	Decrease	29 (76%)	18 (67%)	32 (94%)	
w0 to w9	Increase/maintained	2 (5%)	6 (22%)	1 (3%)	0.020
	Decrease	36 (95%)	21 (78%)	33 (97%)	

* chi^2^ test; w0—baseline; w5—after 4 weeks of intervention, in week 5; w9—after 8 weeks of intervention, in week 9.

## Data Availability

Data is available on request from the corresponding author. The data are not publicly available due to ethical reasons.

## Supplementary Materials

The following supporting information can be downloaded at: https://www.mdpi.com/article/10.3390/nu16203565/s1, Table S1: Detailed analysis of the daily intake of fish species grouped based on their vitamin D content, the daily intake of fish products, and the total daily fish and fish products intake across the different study groups at baseline, during the 4 first weeks and the last 4 weeks of the intervention; Table S2: The number of participants whose 25(OH)D serum concentration increased or was maintained compared to those whose 25(OH)D decreased during the different study periods with both intervention groups combined.
