# Supplementary material for: Effect of Consuming Salmon Products on Vitamin D Status of Young Caucasian Women in Autumn—A Randomized 8-Week Dietary VISA 2 (Vitamin D in Salmon Part 2) Intervention Study
Source: Nutrients. 2024 Oct 21;16(20):3565. doi: 10.3390/nu16203565 (PMC11510608; doi:10.3390/nu16203565)
Supplement: Supplementary file 1 [file nutrients-16-03565-s001.zip › nutrients-3255163-supplementary.pdf]

nutrients

MDPI

Supplementary Materials

Effect of Consuming Salmon Products on Vitamin D Status of Young Caucasian Women in Autumn – a Randomized 8-Week Dietary VISA 2 (Vitamin D In Salmon Part 2) Intervention Study

Zofia Utri-Khodadady, Dominika Głąbska and Dominika Guzek

Table S1. Detailed analysis of the daily intake of fish species grouped based on their vitamin D content, the daily intake of fish products, and the total daily fish and fish products intake across the different study groups at baseline, during the 4 first weeks, and the last 4 weeks of the intervention.

**Daily intake, g/day**			**Smoked salmon intervention group** n=38		**Salmon sausage intervention group** n=27		**Control group** n=34		*p***
			Mean ± SD	Median (P25; P75)	Mean ± SD	Median (P25; P75)	Mean ± SD	Median (P25; P75)	
w0 (baseline)	Fresh and smoked fish	with high vitamin D content	5.3 ± 5.4	3.3 (1.7; 6.7)*	4.7 ± 4.4	3.3 (1.7; 6.7)*	3.7 ± 3.4	3.3 (1.7; 5.0)*	0.421
		with medium vitamin D content	1.8 ± 1.8	1.7 (0.4; 3.3)*	2.6 ± 2.5	1.7 (1.0; 3.3)*	2.1 ± 2	1.7 (0.3; 3.3)*	0.469
		with low vitamin D content	1.7 ± 2.0	1.7 (0.0; 2.9)*	2.6 ± 2.4	2.5 (0.0; 3.3)*	3.1 ± 5	1.7 (0.0; 3.3)*	0.286
		Fish products	5.4 ± 5.4	3.3 (1.9; 6.7)*	8.8 ± 8.1	6.7 (3.3; 13.3)*	6.5 ± 11.8	3.3 (0.0; 9.4)*	0.154
	Total fish and fish products		14.2 ± 8.4	12.5 (8.0; 19.6)*	18.8 ± 10.8	18.3 (10.8; 26.7)	15.5 ± 15	11.3 (7.7; 18.3)*	0.119
w1 to w4 (4 first weeks of intervention)	Fresh and smoked fish	with high vitamin D content	26.0 ± 3.4	23.3 (23.3; 28.3)*[a]	4.1 ± 7.9	0.0 (0.0; 3.3)*[b]	2.6 ± 2.6	3.3 (0.0; 3.3)*[b]	<0.001
		with medium vitamin D content	1.8 ± 2.6	1.7 (0.0; 2.9)*	1.7 ± 2.1	1.7 (0.0; 2.5)*	1.7 ± 2.4	0.0 (0.0; 3.3)*	0.958
		with low vitamin D content	2.0 ± 4.4	0.0 (0.0; 1.7)*	1.9 ± 3.5	0.0 (0.0; 3.3)*	2.3 ± 3.4	0.0 (0.0; 4.6)*	0.771
		Fish products	4.5 ± 6.5	3.3 (0.0; 6.0)*[a]	97.5 ± 4.9	96.7 (93.3; 100.0)*[b]	5.3 ± 7.9	3.3 (0.0; 9.2)*[a]	<0.001
	Total fish and fish products	including intervention	34.3 ± 10.3	31.7 (26.7; 36.3)*[a]	105.0 ± 12.2	102.0 (95.8; 111.0)*[b]	11.8 ± 10.8	9.2 (3.5; 16.3)*[c]	<0.001
		excluding intervention	11.0 ± 10.3	8.3 (3.3; 12.9)*	11.4 ± 11.4	8.3 (2.5; 17.5)*	11.8 ± 10.8	9.2 (3.5; 16.3)*	0.913
w5 to w8 (4 last weeks of intervention)	Fresh and smoked fish	with high vitamin D content	26.4 ± 7.8	23.3 (23.3; 26.7)*[a]	1.6 ± 2.3	0.0 (0.0; 3.3)*[b]	2.8 ± 4.0	1.7 (0.0; 4.6)*[b]	<0.001
		with medium vitamin D content	1.6 ± 2.3	0.0 (0.0; 2.9)*	1.1 ± 2.1	0.0 (0.0; 1.7)*	1.2 ± 2.3	0.0 (0.0; 1.7)*	0.576
		with low vitamin D content	1.0 ± 1.8	0.0 (0.0; 1.7)*	1.3 ± 2.6	0.0 (0.0; 0.8)*	1.5 ± 3.4	0.0 (0.0; 1.7)*	0.908
		Fish products	3.9 ± 6.1	0.0 (0.0; 6.7)*[a]	97.7 ± 6.2	96.7 (93.3; 100.0)*[b]	4.4 ± 5.2	3.3 (0.0; 6.7)*[a]	<0.001

Total fish and fish products	including intervention	32.7 ± 10.4	30.0 (24.4; 38.8)*[a]	102.0 ± 8.3[c]	100.0 (95.0; 107.0)	10.0 ± 8.2	8.3 (3.8; 14.6)*[b]	<0.001
	excluding intervention	9.5 ± 10.4	6.7 (1; 15.4)*	8.5 ± 7.9	6.7 (2.5; 13.3)*	10.0 ± 8.2	8.3 (3.8; 14.6)*	0.719

Fish with high vitamin D content: ≥15 µg/100 g, salmon (*Salmo salar*), rainbow trout (*Oncorhynchus mykiss*), herring (*Clupea harengus*), and eel (*Anguilla anguilla*); fish with medium vitamin D content: 2.1–8.0 µg/100 g, halibut (*Hippoglossus hippoglossus*), mackerel (*Scomber scombrus*), brook trout (*Salvelinus fontinalis*), sole (*Solea solea*), and tuna (*Thunnus*); fish with low vitamin D content: ≤1.0 µg/100 g, cod (*Gadus morhua*), flounder (*Platichthys flesus*), plaice (*Pleuronectes platessa*), pollock (*Gadus chalcogrammus*), hake (*Merluccius merluccius*), perch (*Perca fluviatilis*), zander (*Sander lucioperca*), and pike (*Esox Lucius*) – classification based on VIDEO-FFQ [37]; *non-normal distribution (verified using Shapiro–Wilk test; $p \leq 0.05$); **Kruskall-Wallis ANOVA test; different letters in rows (a, b, c) indicate significant differences between groups ($p<0.05$).

Table S2. The number of participants whose 25(OH)D serum concentration increased or was maintained compared to those whose 25(OH)D decreased during the different study periods with both intervention groups combined.

**Time**	**25(OH)D serum concentration change, nmol/l**	**Intervention groups (smoked salmon + salmon sausage)** n=65	**Control group** n=34	***p****
w0 to w5	Increase/ maintained	14	3	0.189
	Decrease	51	31	
w5 to w9	Increase/ maintained	18	2	0.021
	Decrease	47	32	
w0 to w9	Increase/ maintained	8	1	0.241
	Decrease	57	33	

[*] chi^2 test with Yates' correction; w0—baseline; w5—after 4 weeks of intervention, in week 5; w9—after 8 weeks of intervention, in week 9.

Disclaimer/Publisher's Note: The statements, opinions and data contained in all publications are solely those of the individual author(s) and contributor(s) and not of MDPI and/or the editor(s). MDPI and/or the editor(s) disclaim responsibility for any injury to people or property resulting from any ideas, methods, instructions or products referred to in the content.
